# Chest Pain: Wellens Syndrome Due to Spontaneous Dissection of the Left Anterior Descending Coronary Artery — A Case Report and Literature Review

**DOI:** 10.31083/j.rcm2502070

**Published:** 2024-02-20

**Authors:** Giuseppe Clemente, Cosimo Quaranta, Maria Grazia Basso, Chiara Pintus, Giuliana Rizzo, Celeste Vullo, Silvia Bruno, Francesca Castro, Danilo Puccio, Roberto Nola, Giuseppina Novo, Egle Corrado, Antonino Tuttolomondo

**Affiliations:** ^1^Internal Medicine and Stroke Care Ward, University Hospital Policlinico P. Giaccone, 90127 Palermo, Italy; ^2^Coronary Intensive Care Unit, University Hospital Policlinico P. Giaccone, 90127 Palermo, Italy

**Keywords:** chest pain, electrocardiographic patterns, spontaneous left anterior descending coronary artery dissection, Wellens syndrome, Wellens pattern Type A and Type B, acute coronary syndrome

## Abstract

Wellens syndrome is an abnormal electrocardiographic pattern characterized by biphasic (type A) or deeply inverted (type B) T waves in leads V2–V3. It is typically caused by temporary obstruction of the left anterior descending (LAD) coronary artery due to the rupture of an atherosclerotic plaque leading to occlusion. Spontaneous coronary artery dissection (SCAD) is a rare cause of acute coronary syndrome and even a rarer cause of Wellens Syndrome. It occurs when an intramural hematoma forms, leading to the separation of the tunica intima from the outer layers and creating a false lumen that protrudes into the real lumen, ultimately reducing blood flow and thus resulting in myocardial infarction. Here we report a case of SCAD presenting as an acute coronary syndrome with self-resolving chest pain, slightly elevated myocardial necrosis markers and electrocardiographic changes consistent with Wellens pattern type A first, and type B afterwards, that were not present upon arrival to the emergency department.

## 1. Introduction

Wellens syndrome, first described in 1982, is an abnormal electrocardiographic pattern characterized by deeply inverted or biphasic T waves in leads V2–V3 with cardiac biomarkers usually normal or slightly elevated. It is typically caused by a temporary obstruction of the left anterior descending (LAD) coronary artery due to the rupture of an atherosclerotic plaque leading to occlusion, with subsequent clot lysis or other disruptions responsible for critical blood flow reduction that may cause massive myocardial infarction (MI) of the anterior wall. The risk factors for Wellens syndrome are similar to those for traditional coronary artery disease, including dyslipidemia, hypertension, diabetes, sedentary lifestyle, obesity, smoking, and metabolic syndrome.

The clinical presentation of patients with Wellens syndrome typically includes symptoms consistent with acute coronary syndrome (ACS), such as tightness or pressure-like chest pain that may radiate to the neck, jaw, or shoulder. However, upon presentation to the emergency department, patients may be pain-free.

The exact mechanism behind the electrocardiogram (ECG) changes in Wellens syndrome is unknown, but some theories suggest that coronary artery spasm and stunned myocardium may be responsible.

**Fig. 1.**
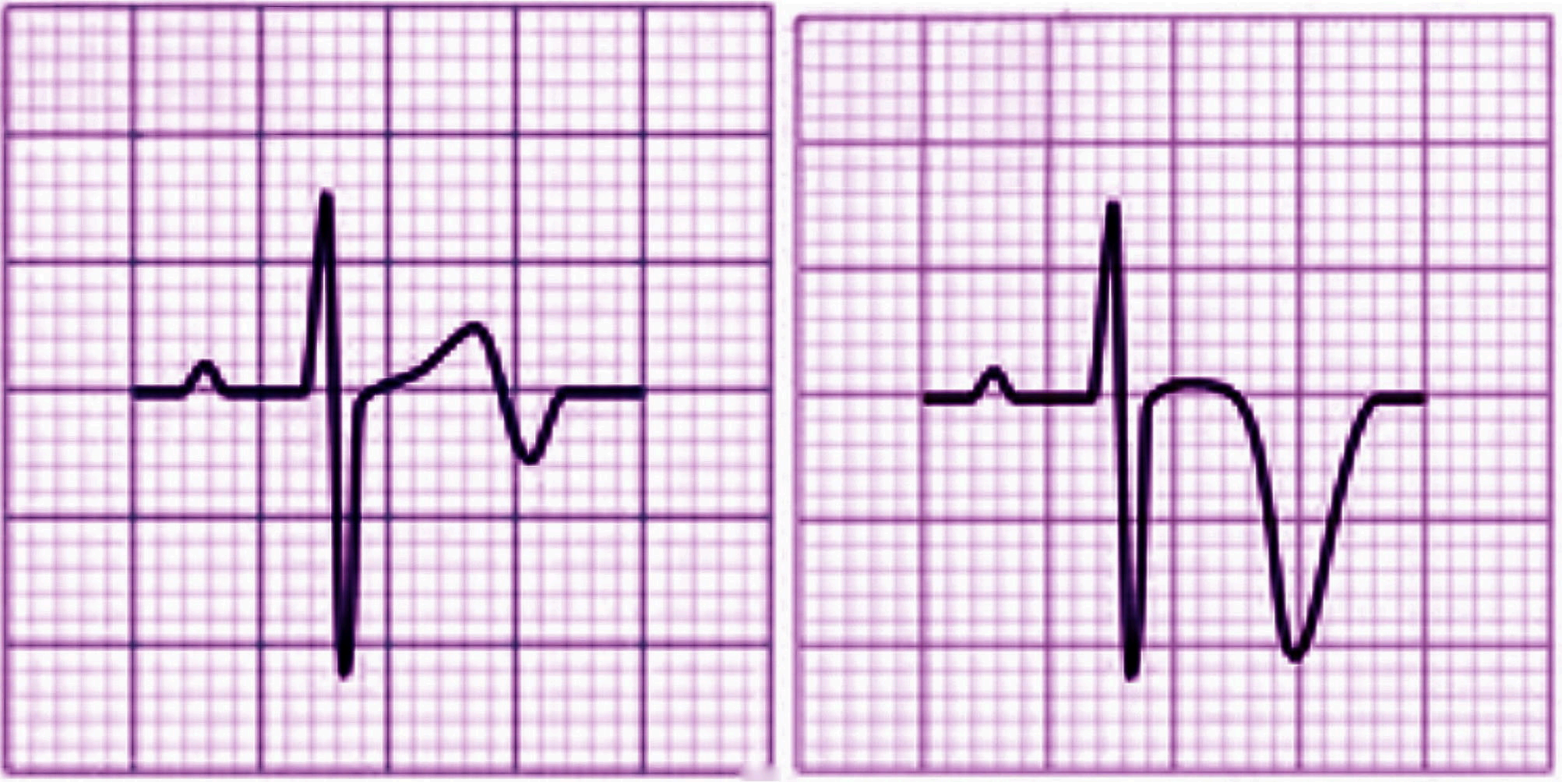
**Electrocardiographic (ECG) changes in Type A (left) and Type B (right) Wellens syndrome**.

There are two patterns of ECG alteration in Wellens syndrome: type A and type B. Type A, which accounts for 25% of cases, is characterized by biphasic T waves with initial positivity and terminal negativity in leads V2 and V3. Type B, on the other hand, accounts for 75% of cases and is characterized by deeply and symmetrically inverted T waves in leads V2 and V3 (Fig. 1). Despite the differences in the ECG patterns, the progression from type A to type B pattern may occur as a consequence of ischemic damage progression, as proposed by S. W. Smith in his book *“The ECG in acute MI” * [1].

The T wave abnormalities associated with Wellens syndrome may persist for hours, up to weeks, even in the absence of symptoms. In some cases, the T wave pattern may even appear to normalize into hyperacute upright T waves, which is known as pseudo-normalization [1, 2].

While the Wellens pattern is specific for stenosis of the LAD coronary artery caused by an occlusive plaque, there are also mimics of Wellens syndrome, referred to as pseudo-Wellens syndrome. These can be caused by various factors such as cocaine and marijuana use, myocardial bridging, and Takotsubo cardiomyopathy [3].

The definitive treatment for Wellens syndrome is cardiac catheterization with percutaneous coronary intervention (PCI). Until this can be performed, the therapeutic strategy is similar to that for acute myocardial infarction (AMI), including antiplatelet therapy, anticoagulation, nitrates, and beta-blockers. It is important to note that patients with Wellens syndrome have little benefit from medical management alone, and procedural intervention is necessary for definitive treatment [4].

## 2. Case Report 

Here we describe the case of a 44-year-old woman who presented to the emergency department with severe, sub-continuous pain localized to the left hemithorax, which radiated to the back, ipsilateral forearm, and jaw and arose after minor exertion. The pain was exacerbated by breathing and was sensitive to acupressure (Chest Pain Score: 8; Heart Score: 4).

The patient denied ischemic equivalents such as dyspnea, lipothymia, syncope, sweating, nausea, and vomiting, but had a history of atypical chest pain with similar symptoms that had appeared five months prior. Since then, she reported frequent episodes, especially during night rest. She was taking nonsteroidal anti-inflammatory drugs (NSAIDs, ketoprofen) at home, suspecting osteoarticular pain, without experiencing any benefit. Her medical history was remarkable for hepatitis C virus (HCV)-related liver disease, while her surgical history was significant for thyroidectomy, cholecystectomy, and appendectomy.

Upon examination, the patient was alert, well-oriented, and cooperative. She was eupneic at rest, with a slightly elevated heart rate (HR: 102 bpm) while the other vital signs were within normal range, and her physical examination was unremarkable.

**Fig. 2.**
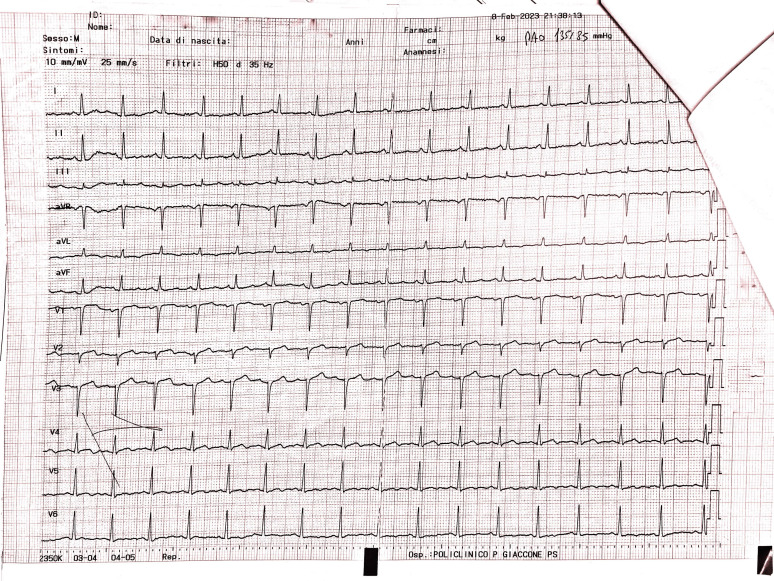
**Admission ECG showing sinus tachycardia with a heart rate of 103 beats per minute**. Poor progression of the R wave in the precordial leads was observed along with minimal ST segment elevation from V1 to V3 in the context of widespread ventricular repolarization abnormalities. ECG, electrocardiogram.

The ECG conducted in the emergency department (Fig. 2 — admission ECG) revealed sinus tachycardia with a heart rate of 103 beats per minute. Poor progression of the R wave in the precordial leads was observed along with minimal ST segment elevation from V1 to V3 in the context of widespread ventricular repolarization abnormalities.

**Fig. 3.**
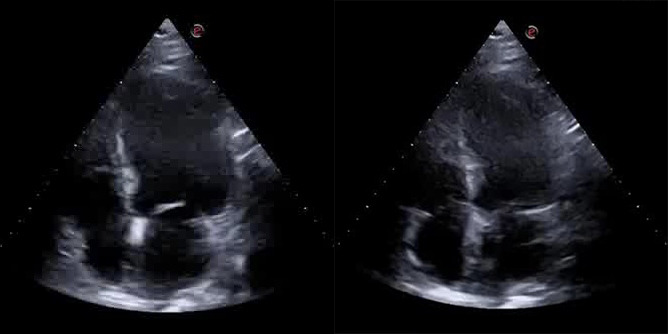
**End-diastolic frame (Left) and End-systolic frame (Right) of the bedside cardiac ultrasound showing evidence of hypo-akinesia in the apex, periapical segments, mid anteroseptal segment and the anterior wall, leading to a mild decrease in overall systolic function (left ventricular ejection fraction [LVEF]: 43%)**.

A bedside cardiac ultrasound was then performed, which showed evidence of akinesia in the apex and periapical segments, the mid anteroseptal segment, and the anterior wall, and hypokinesia in the mid anterolateral segment, leading to a mild decrease in overall systolic function (left ventricular ejection fraction [LVEF]: 43%). The left ventricle was slightly dilated with a minor increase in parietal thickness and a II degree diastolic dysfunction. Additionally, there was mild enlargement of the left atrium, mild-to-moderate mitral regurgitation, and mild tricuspid insufficiency with an estimated pulmonary arterial pressure systolic (PAPs) of 45 mmHg (Fig. 3).

The three hour high sensitive troponin T delta was negative but there was a high value at time zero ($T_{0}$: 144 ng/L–$T_{1}$: 175 ng/L), with renal function tests within normal range.

Another noteworthy laboratory finding was evidence of hypochromic microcytic anemia (Hemoglobin [Hb] 8.2 g/dL, Hematocrit (Hct) 30.3%, mean corpuscular volume (MCV) 68.6 fL, mean corpuscular hemoglobin (MCH) 18.6 pg).

Given the complete resolution of chest pain and the absence of additional ischemic equivalents, the patient was admitted to the Department of Internal Medicine for Cardiovascular investigation, where she underwent clinical and ECG monitoring.

**Fig. 4.**
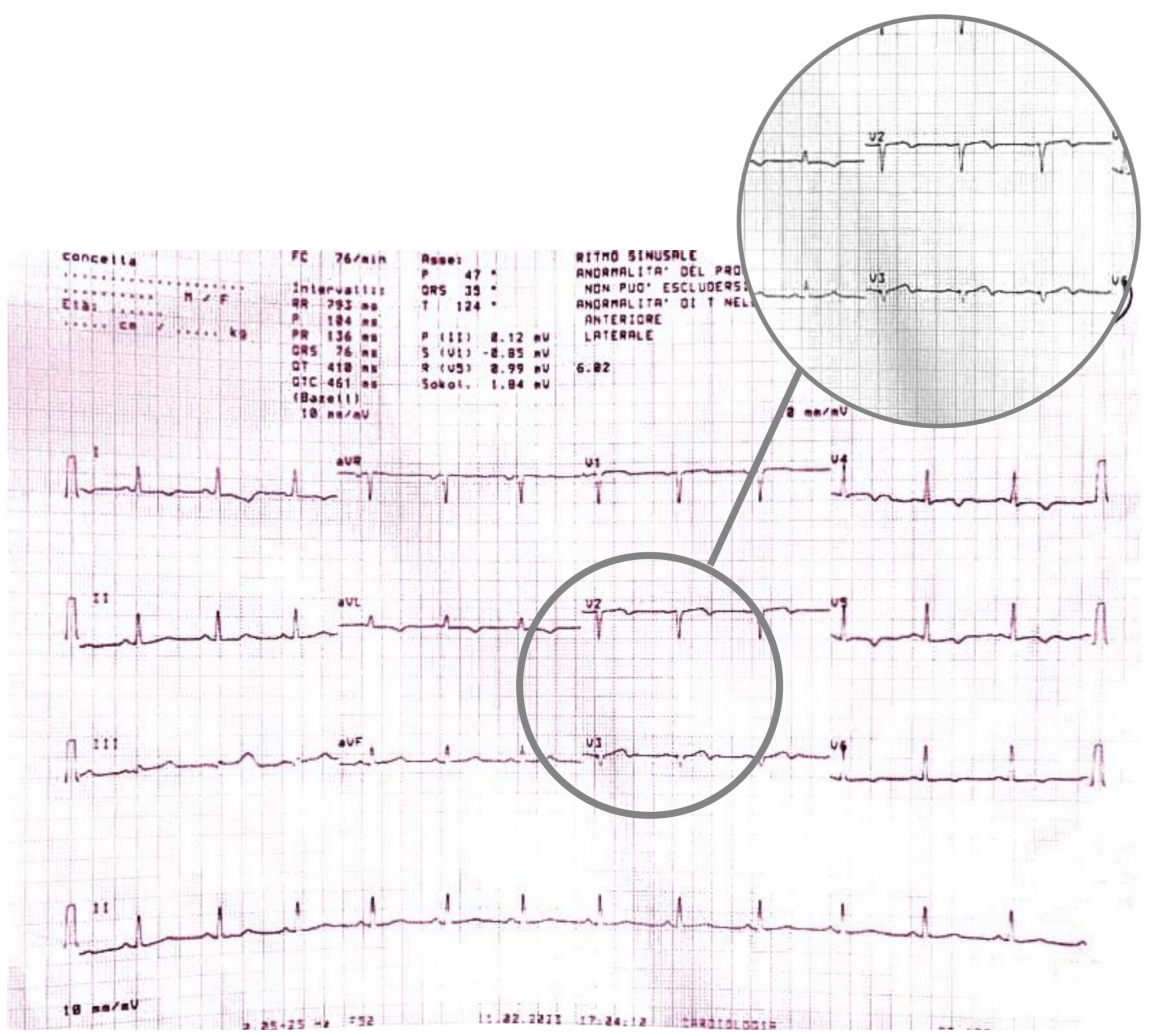
**ECG showing biphasic T waves with initial positivity and terminal negativity in leads V2 and V3 slightly resembling type A Wellens Syndrome**. ECG, electrocardiogram.

During the hospitalization, the patient experienced the recurrence of similar episodes of mild chest pain; all of them occurred after minor exertion, lasted a few minutes and were self-resolving. The 12 leads ECG performed during the last one of them showed few changes: biphasic T waves with initial positivity and terminal negativity in leads V2 and V3 slightly resembling type A Wellens pattern were present (Fig. 4 — ECG).

**Fig. 5.**
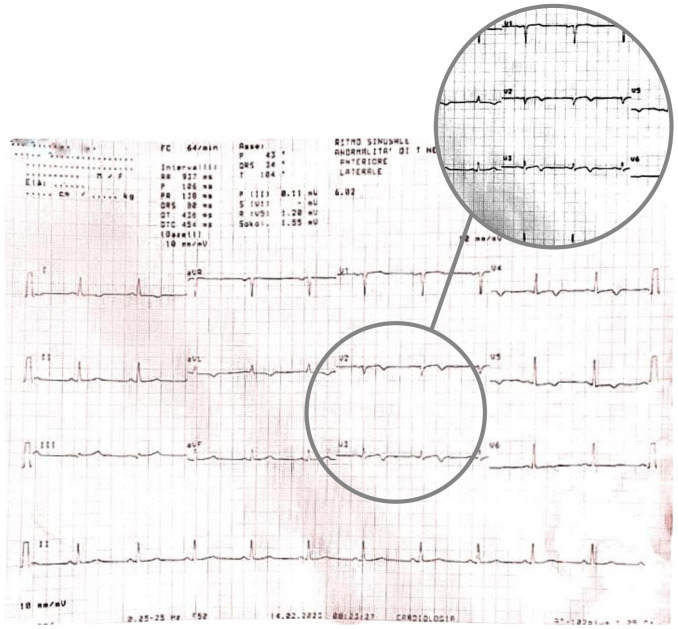
**ECG showing symmetrically inverted T waves in leads V2 and V3, resembling a type B Wellens pattern**. ECG, electrocardiogram.

A few minutes later, the patient experienced a further episode of chest pain, which occurred at rest, but was of greater intensity and duration, and had no clear triggering factors, another 12-lead ECG was performed and showed some differences compared to previous recordings: symmetrically inverted T waves were evident in leads V2 and V3, this time resembling a type B Wellens pattern (Fig. 5 — ECG). As discussed above, these ECG patterns, in a patient admitted for chest pain with slightly elevated serum cardiac markers and negative 3-hour hs-troponin T delta, should raise suspicion of Wellens Syndrome, which is highly specific for critical stenosis of the LAD.

The Cardiac Intensive Care Unit (CICU) was promptly notified, and the transfer was arranged accordingly. In the meantime, an additional hs-troponin test was performed, and a bedside echocardiogram was carried out, which confirmed the akinesia in the apex and in the septal segments. These myocardial territories are supplied by the left anterior coronary artery, thus further corroborating the diagnosis.

**Fig. 6.**
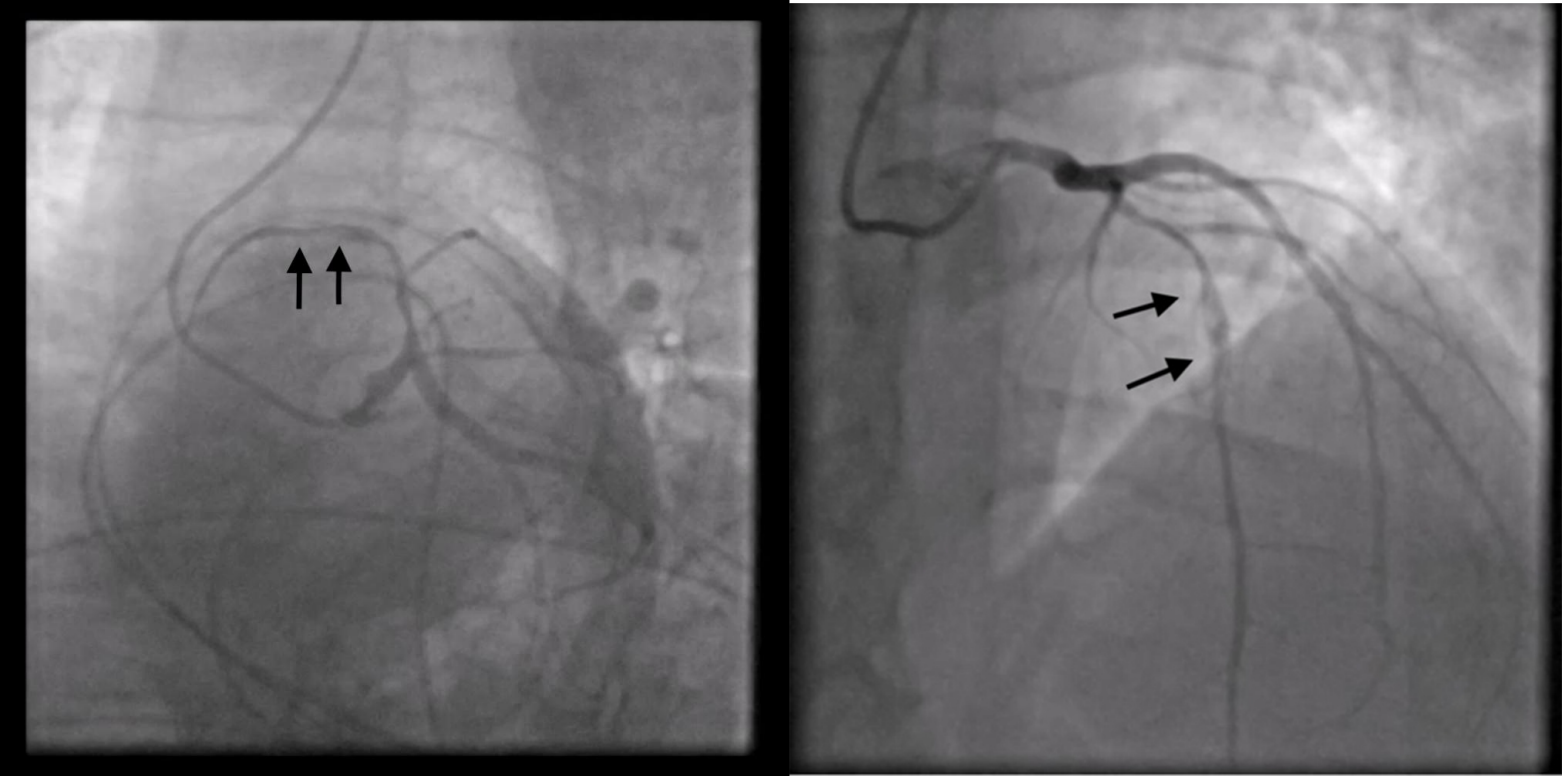
**Coronary angiography shows no stenosis; instead, it reveals a spontaneous type 2 dissection of left anterior descending (LAD) coronary artery**.

The new hs-troponin test results, showing a new peak of myocardionecrosis markers of 289 ng/L, came when the patient had already been transferred to the CICU, where, finally, an emergency coronary angiography (CAG) was performed. Surprisingly enough, the CAG showed no stenosis; instead, it revealed a spontaneous dissection evident in the middle tract of the LAD coronary artery; this was classified as a type 2 dissection of the LAD coronary artery according to the European Society of Cardiology (ESC) classification (Fig. 6). The angiographic images show a plausible proximal extension of the dissection, where the vessel appears diffusely reduced in caliber; however, the same findings could also be indicative of diffuse atherosclerotic disease in the proximal LAD artery. In the acute setting, given the high suspicion of proximal extension, it was not deemed appropriate to use intracoronary imaging techniques such as intravascular ultrasound (IVUS) or optical coherence tomography (OCT) because of the associated risks of catheter-induced dissection propagation, potentially aggravating the patient’s condition and endangering her life.

Consequently, the patient was diagnosed with AMI and spontaneous LAD coronary artery dissection and was then promptly transferred to our institution’s cardiology department for further evaluation and management.

Upon admission, the patient’s blood pressure was 135/80 mmHg, with a heart rate of 71 bpm. The ECG displayed sinus rhythm, anteroseptal necrosis, negative T waves in leads DI, aVL, V4, V5, and V6, along with isodiphasic T waves in leads V2 and V3. Laboratory tests also revealed elevated levels of N-terminal pro-B-type natriuretic peptide (NT-proBNP) at 1949 ng/L, and low-density lipoprotein (LDL) at 114 mg/dL.

Echocardiographic examination revealed a mildly dilated left ventricle with normal parietal thickness. Akinesia in the apex, periapical segments, middle septum, and middle anterior wall was also noted. Additionally, there was a moderately depressed global systolic function, with an ejection fraction of 40%. The exam also showed moderate mitral valve insufficiency.

Considering the patient’s hemodynamic and electrical stability, and in line with the latest evidence-based literature, a conservative treatment approach was chosen. The prescribed treatment regimen included aspirin, an angiotensin converting enzyme (ACE)-inhibitor, a beta-blocker, a mineralocorticoid receptor antagonist (MRA), and a statin.

**Fig. 7.**
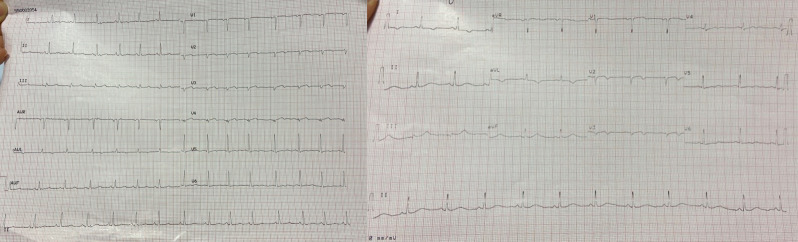
**A re-evaluation of the ECG during episodes of acute chest pain revealed isodiphasic T waves in leads V2 and V3**. ECG, electrocardiogram.

During the patient’s hospital stay, she experienced several episodes of acute chest pain. A re-evaluation of the ECG during these episodes revealed isodiphasic T waves in leads V2 and V3 (Fig. 7).

**Fig. 8.**
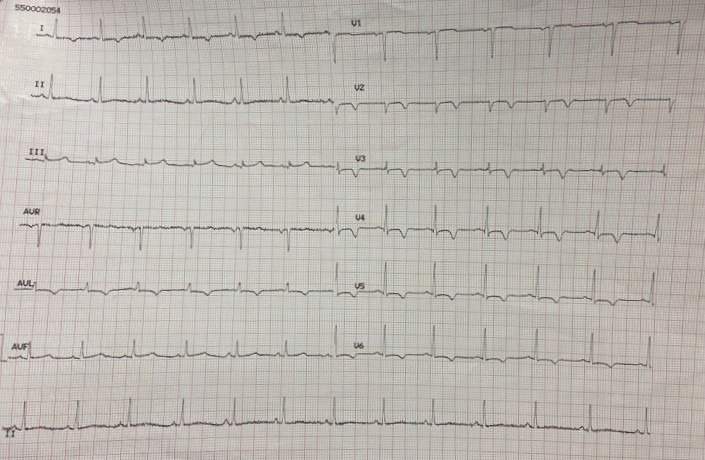
**ECG at rest, in the absence of symptoms, displays negative T waves in the extended-anterior region**. ECG, electrocardiogram.

When the ECG was recorded at rest, without symptoms, it displayed negative T waves in the extended-anterior region (Fig. 8).

After a week of receiving symptomatic treatment, the patient’s condition improved, and she no longer experienced chest pain. To further investigate potential sites of dissection, aneurysm, or intramural hematoma, she underwent a comprehensive computed tomography (CT) angiography with and without contrast. The results did not reveal any significant pathological alterations. However, on the same day, the patient decided to discharge herself against medical advice.

A computed tomography coronary angiography (CTCA) was scheduled three months after discharge to assess the healing of the spontaneous coronary artery dissection (SCAD) and to clarify the etiology of the proximal LAD constriction: proximal extension of the dissection or diffuse atherosclerotic disease?

## 3. Coronary Artery Dissection — A Review of the Literature

### 3.1 Spontaneous Coronary Artery Dissection

**Fig. 9.**
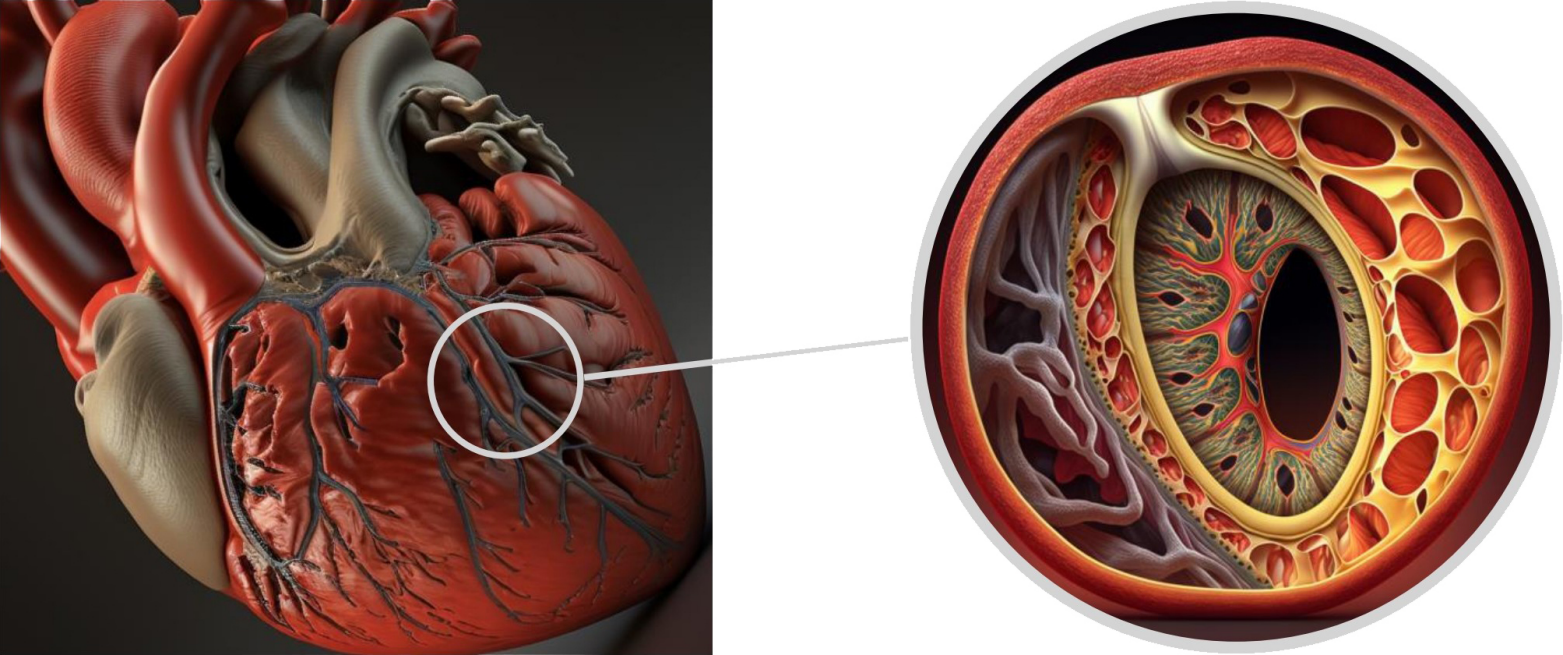
**Tridimensional representation of LAD coronary artery dissection. **LAD, left anterior descending.

The arterial wall is composed of three distinct layers. The innermost layer, known as the tunica intima, consists mainly of endothelial cells and connective tissue. The middle layer, called the tunica media, is comprised of smooth muscle cells, while the outer layer, the tunica adventitia, is made up of collagen and elastic fibers and includes the vasa vasorum [5]. Coronary dissection occurs when an intramural hematoma forms, leading to the separation of the tunica intima from the outer layers and creating a false lumen that protrudes into the real lumen, ultimately reducing blood flow (Fig. 9).

### 3.2 Epidemiology and Risk Factors

Spontaneous coronary artery dissection (SCAD) is a relatively rare etiology of ACS, accounting for approximately 4% of all MIs [6, 7, 8]. It primarily affects the LAD artery, while other branches and rami of the coronary circulation are less commonly involved [9].

Despite its severity, SCAD is associated with low mortality rates, estimated at around 1–2% [10]. However, the incidence of SCAD is known to increase in specific populations, such as young women under 50 years old or pregnant women with ACS, who do not exhibit conventional cardiovascular risk factors. Conversely, men are rarely affected [11, 12]. In these populations, the prevalence of SCAD can reach as high as 45.0% and 43%, respectively [13, 14, 15, 16, 17, 18].

Several predisposing conditions have been identified in SCAD development, such as pregnancy, fibromuscular dysplasia, and genetic susceptibility. These conditions can be exacerbated by precipitating factors, such as sex hormones, emotional stress, and certain medications. SCAD’s higher incidence in women and its association with pregnancy suggests a significant role for sex hormones in its pathophysiology. These hormones may weaken connective tissue and the microvascular system, increasing the risk of vessel wall rupture and/or intramural hematoma formation [19, 20, 21]. Changes in circulating estrogen and progesterone levels appear to underpin SCAD’s pathophysiological mechanism, though the specifics remain unclear. Pregnancy-associated SCAD (P-SCAD) typically occurs in the postpartum period, generally within the first two weeks. P-SCAD patients often present with severe clinical manifestations, including shock, left ventricular dysfunction, multivessel dissection, and involvement of the interventricular artery. Nearly half of P-SCAD cases exhibit ST-segment elevation, and P-SCAD represents between 5–17% of all SCAD cases. Women with P-SCAD tend to be older at first childbirth and multigravidas, often with a history of fertility treatments or pre-eclampsia [22, 23, 24, 25].

Studies have revealed an association between a common non-coding variant (rs9349379) in the *PHACTR1/EDN1* locus, a genetic locus associated with other pathologies such as fibromuscular dysplasia, and an increased risk of developing SCAD [21, 26]. Additional genes are likely implicated in SCAD development, and research in this area is ongoing [22].

First described in 1938, fibromuscular dysplasia (FMD) affects the medium-sized artery wall and leads to the formation of aneurysms or dissection. The pathogenesis of FMD is still unknown, although a genetic substrate has been proposed, but no single genetic mutation has been identified [27]. FMD represents a risk factor for SCAD: 10.5% of patients with FMD had an arterial dissection, and 2.5% had SCAD [28]. On the other hand, 31.1% to 45.0% of patients with SCAD are diagnosed with underlying FMD; for this reason, all patients with SCAD should be screened for this disease [29]. Post-hospitalization, patients should be advised to undergo CTCA or magnetic resonance angiography to evaluate the vasculature of the head, neck, abdomen, and pelvis [30]. This is of utmost importance since it is the arteriopathy most commonly associated with SCAD and it usually occurs in middle-aged women with few cardiovascular risk factors [31].

Other connective disorders, such as Marfan syndrome, Ehlers–Danlos syndrome, or Loeys–Dietz syndrome, and systemic inflammatory diseases are not significantly associated with SCAD.

Environmental stressors and extreme physical exercise may also be related to SCAD, suggesting that the hyperactivation of the adrenergic system may be a pathogenic substrate for the disease [32]. Indeed, previous physical exertion and emotional stress have been identified in 40% and 24% of SCAD patients, respectively [10, 33, 34].

### 3.3 Pathophysiology

SCAD is a non-atherosclerotic phenomenon characterized by the formation of a false lumen filled with blood, which consequently compresses the true lumen, thereby compromising blood flow [22, 31].

Two primary theories have been postulated to explain this process. The ‘inside out’ hypothesis posits that blood permeates the subintimal space via an intimal tear or flap, while the ‘outside in’ hypothesis attributes the formation of a media hematoma to a rupture of the vasa vasorum [22]. The latter appears to be the more prevalent mechanism, as early SCAD angiograms typically reveal an intramural hematoma without intimal disruption [35].

Intracoronary OCT studies do not show any communication between the false and true lumens. OCT does, however, highlight the pressurization of the false lumen, which can extend the intramural hematoma and exacerbate stenosis in non-fenestrated cases. Conversely, in fenestrated instances, the differential pressure between lumens can lead to intimal rupture [36]. Though most SCAD patients exhibit flow obstruction, arteries can occasionally appear normal or show gradual narrowing, prompting a diagnosis of myocardial infarction with nonobstructive coronary arteries (MINOCA) [20].

Lastly, it is worth mentioning that, although atherosclerosis is the primary etiology of non-spontaneous coronary artery dissection, the literature does describe rare cases where concomitant atherosclerotic pathology and spontaneous coronary dissection co-occur [37].

### 3.4 Clinical Features

Intramural hematoma formation and the expansion of the false channel along the longitudinal axis can obstruct blood flow and cause myocardial ischemia. SCAD is a rare cause of ACS, therefore, the most common presentation includes acute chest pain and angina equivalents, accompanied by primary ECG alterations of ventricular repolarization (such as ST-segment elevation or non-ST-segment elevation, T-wave inversions), and elevated cardiac biomarkers in most cases. However, in some cases, 0.4% to 4.0% of patients may present with normal troponin values [38, 39, 40]. Less frequent symptoms may include back pain (14%), shortness of breath (20%), and unspecific symptoms such as nausea, vomiting (24%), diaphoresis (21%), or dizziness (9%) [41]. Interestingly enough, following the admission of patients to hospital, ongoing symptoms of chest pain are not due to myocardial ischemia and rather represent pain due to extending coronary dissection, thus it is of utmost importance to treat the patients appropriately with analgesia and strict blood pressure control [21].

Less frequently, SCAD may present with ventricular arrhythmias, sudden cardiac arrest, or papillary muscle rupture, leading to acute congestive heart failure and cardiogenic shock. Physical examination, in this case, is characterized by the presence of lower limb pitting edema, jugular vein turgor, crepitations in the lung, sinus tachycardia, and S3 gallop rhythm [40]. Complications are more prevalent in peripartum patients.

Due to the non-specificity of the clinical presentation, an angiographic study is required to confirm the diagnosis of SCAD.

### 3.5 Diagnosis

CAG is the principal diagnostic tool that, in most instances, enables the detection and assessment of SCAD’s extent. Intracoronary imaging should be considered when diagnosis is uncertain or if an invasive therapeutic approach is required [31, 42].

SCAD manifests in a variety of angiographic forms. Saw *et al*. [43] delineated three types of angiographic presentations, with Al-Hussaini and Adlam [44] subsequently contributing a fourth. The current classification, endorsed by the ESC [22, 31], recognizes four main types:

Type 1: Characterized by a radiolucent flap and a double-track image owing to contrast stagnation in the false lumen. Although this is SCAD’s pathognomonic pattern and is relatively easy to identify, it only accounts for 29% of cases.

Type 2: Features a pronounced narrowing of the vessel lumen, typically >20 mm, induced by compression from an intimal hematoma (which can be visualized via intracoronary imaging). This pattern is the most prevalent, accounting for about 67% of SCAD cases. It is further divided into:

Type 2a: Denotes the distal restoration of the coronary vessel’s native caliber.

Type 2b: Refers to the extension of the intramural hematoma up to the distal end of the vessel, culminating in a terminal “rat tail” appearance.

Type 3: Displays focal narrowing of the lumen (>20 mm), indistinguishable from an atherosclerotic lesion, and comprises only 4% of cases. It is the most challenging to diagnose due to its mimicry of the atherosclerotic process, necessitating intravascular imaging frequently.

Type 4: Involves total occlusion of the vessel, typically distal, and can resemble a thrombotic occlusion, thus complicating its diagnosis.

The literature describes a few cases of dissection extending proximally, which may jeopardize blood flow in vessels proximal to the lumen of the initially involved coronary artery, potentially necessitating emergency surgical intervention [45].

Additional angiographic elements can aid in SCAD identification, such as coronary tortuosity, the start or end of the false lumen at a side branch, the presence of coronary FMD, the absence of atherosclerosis in other coronaries, and association with myocardial bridging. The “stick insect” sign, a narrowing due to extrinsic compression by an intramural hematoma (IHM) with a biconcave lumen appearance, often interrupted by a side branch, and the “radish appearance”, a distal occlusion due to IHM compression, are characteristic signs [31, 46].

While SCAD can affect any coronary artery, it most frequently involves the anterior descending artery. Typically, the mid-distal coronary tract is affected [21, 42], with a type 2 presentation [44]. In cases of proximal involvement, type 1 is the most common [44]. In 10–15% of cases, dissection occurs concurrently in multiple arteries without continuity [31, 42].

IVUS facilitates differentiation between stenosis resulting from atherosclerotic plaque and SCAD. Also, it can identify false and true lumina, the presence and extent of an intramural hematoma [47]. IVUS-enhanced ultrasound penetration allows for comprehensive visualization of the vessel wall up to the external elastic lamina and superior characterization of the thrombus. However, its poor spatial resolution may fail to highlight all SCAD-related structures or abnormalities, such as the intimal-medial membrane and focal interruptions connecting the false and true lumina [31, 39, 44].

OCT enhances the identification of true and false lumina (assessing size, longitudinal and circumferential extent), offers superior visualization of the intimal-medial membrane, showing lumen compression or enlargement of media/adventitia causing compression, and may detect the presence of an intimal tear (the entry point) [31, 48]. Therefore, OCT is highly beneficial for confirming or refuting the diagnosis when angiographic images suggest SCAD. Both IVUS and OCT can guide revascularization procedures in patients when necessary, ensuring the guidewire’s correct positioning in the true lumen and thus, accurate stent placement [31, 48]. Furthermore, OCT allows for the assessment of the actual dimensions (diameter and length) of the segment requiring treatment. In combination with the co-registration technique, this can help prevent mispositioning or misalignment of the stent, which could exacerbate the intramural hematoma or dissection line [49]. Additionally, intracoronary imaging (particularly OCT) allows for an evaluation of stent adherence to the vessel walls and the vessel’s characteristics post-stenting [47].

However, it is essential to remember these methods are not without potential complications, such as catheter-induced dissection propagation or contrast medium-induced ischemia during OCT. Therefore, their routine use is not typically recommended [21, 31].

In cases where diagnostic uncertainty persists, CTCA and cardiac magnetic resonance (CMR) may be used, though their diagnostic accuracy is lower, particularly during the acute phase. CTCA enables the “triple rule-out” of chest pain and offers the advantage of being non-invasive [50]. Specific features have been identified through CTCA: abrupt luminal stenosis, intramural hematoma, tapered luminal stenosis, and dissection [51]. However, its poor spatial resolution and reported false negatives cases (for instance, where intramural hematoma appears similar to noncalcified atherosclerotic plaque or an artifact) [51, 52] do not make it the exam of choice. Nonetheless, CTCA may prove useful for follow-up assessments [31, 50].

In patients with SCAD, CMR may reveal abnormal wall motion, edema, abnormal perfusion, microvascular obstruction, and late gadolinium enhancement in the affected coronary territory, all of which indicate infarction [53, 54]. CMR can help confirm the diagnosis or suggest an alternative diagnosis in patients with unclear etiology of ACS [52].

### 3.6 Differential Diagnosis 

Differentiating between SCAD and other conditions such as atherosclerotic ACS, vasospasm, Takotsubo syndrome, and coronary thromboembolism is of paramount importance [31]. In particular, atherosclerotic ACS can present similarly to SCAD. It may manifest as Type 3 SCAD or mimic Type 1 SCAD following thrombus recanalization. Atherosclerotic ACS is predominantly observed in older patients and males, with a high prevalence of cardiovascular risk factors. Much like SCAD, Takotsubo syndrome is more prevalent in women and shares similar clinical characteristics, often preceded by psychosocial or emotional stress. However, it does not exhibit typical angiographic findings [55].

### 3.7 Therapeutic Strategies 

The therapeutic approach to SCAD is highly personalized and heavily influenced by the patient’s unique clinical presentation and comorbidities [56].

Predominantly, a conservative approach to SCAD treatment is recommended, particularly when the patient is hemodynamically stable, distal blood flow is preserved, and no signs of progressive myocardial ischemia are apparent [19]. This approach is effective in managing uncomplicated SCAD in up to 80% of cases, given the condition’s self-limiting and self-healing nature. Notably, revascularization procedures do not seem to prevent recurrence [31].

The role of antiplatelet therapy in SCAD treatment has been a long-standing topic of discussion. Single anti-platelet therapy has been considered a potential treatment option. According to the European multicenter DISCO registry (DIssezioni Spontanee COronariche [DISCO] multicentre international registry), dual antiplatelet therapy (DAPT) appears to be associated with a higher incidence of cardiovascular events at one-year follow-up compared to single antiplatelet therapy, thus establishing the latter as the preferred treatment option [57].

The early initiation of anticoagulant therapy could potentially alleviate thrombotic burden. However, it may also extend the dissection, hence routine anticoagulant therapy is typically not recommended unless clear indications like an intraluminal thrombus or other systemic anticoagulation indications are present [21].

The use of glycoprotein IIb/IIIa inhibitors in SCAD management is not recommended [21]. Conversely, beta-blockers are advocated owing to their potential to reduce the risk of recurrence. By decreasing myocardial contractility, heart rate, and blood pressure, beta-blockers can reduce wall stress, making them a crucial component in aortic dissection management [10].

Saw *et al*. [10] were pioneers in suggesting that beta-blocker therapy could reduce the risk of recurrent dissection. This study also examined the correlation between hypertension and recurrent SCAD, noting that systemic hypertension increases wall stress by prompting arterial remodeling, which could theoretically increase the risk of arterial dissection. Therefore, rigorous blood pressure management is essential in both acute and long-term SCAD management [10].

Angiotensin-converting enzyme inhibitors (ACEIs) and mineralocorticoid receptor antagonists (MRAs) have broader applications in managing patients with both ST-elevation myocardial infarction (STEMI) and non-ST-elevation ACS (NSTE-ACS) and heart failure with a reduced ejection fraction [10, 31].

Depending on the clinical scenario, arterial vasodilators like nitrates and calcium antagonists can be administered. However, their routine administration is not typically recommended [31]. In cases where chest pain persists in patients unsuitable for revascularization therapy, or signs of coronary vasospasm or microvascular dysfunction are present, nitrates, calcium antagonists, or ranolazine could be considered [21].

The use of statins is dictated in the presence of concurrent conditions that warrant their application. A study published in 2012 investigated the association between statin use and SCAD recurrence, finding a higher incidence of recurrence in the group prescribed statins. However, because the median year of the index event was 2007 for those prescribed statins, compared to 2002 for those not prescribed statins, the date of the event was considered a potential confounding factor. [40]. Nevertheless, routine statin therapy is not commonly advised [31].

Thrombolysis is contraindicated in SCAD due to the risk of promoting dissection propagation, potentially leading to coronary rupture and cardiac tamponade [58].

Revascularization therapy is rarely recommended [59]. PCI for SCAD is more likely to fail than PCI for atherosclerosis-related MI, with potential complications such as wire insertion into the dissection’s false lumen, dissection expansion, and hematoma extension [58].

Certain high-risk patients, identified by persistent angina, ongoing ST-segment elevation, hemodynamic or electrical instability, multiple proximal dissections, left main coronary artery dissection, or thrombolysis in myocardial infarction (TIMI) 0 and 1 coronary flow, may require consideration for percutaneous or surgical revascularization. For percutaneous revascularization, the procedure’s feasibility is determined by the dissection site and coronary anatomy [31, 59].

In SCAD, the primary objective of PCI is to restore flow rather than to resolve the dissection, as most cases spontaneously heal. Hence, a minimalist approach is critical [31]. However, the efficacy of percutaneous intervention in improving outcomes remains unproven [30].

Aortocoronary bypass bears a higher procedural risk and does not confer long-term benefits due to the frequent occurrence of functional bypass occlusion after the dissection heals. While graft failure on follow-up is common, coronary artery bypass grafting (CABG) can limit the extent of myocardial damage during AMI [31]. Although rare, the risk of internal mammary artery dissection should be kept in mind when CABG is considered [60]. When aortocoronary bypass is deemed necessary, the use of venous conduits is recommended due to the high rate of long-term functional occlusion [31].

A small percentage of patients treated conservatively may experience early complications, most commonly due to dissection extension within the first week following the acute episode. Consequently, it is advisable to monitor patients in a hospital setting for at least one week. Generally, spontaneous recovery is observed within approximately one month [21, 61].

### 3.8 Follow-up

Similar to other causes of MI, SCAD necessitates a thorough evaluation, typically employing colordoppler echocardiogram or cardiac magnetic resonance imaging (MRI) around three months post the index event, in order to assess left ventricular systolic function, a critical step in guiding subsequent medical and potential device therapy.

Given the close association with fibromuscular dysplasia, a comprehensive imaging strategy of extracoronary vascular districts using CT, MRI, or peripheral angiographic study is advised. Currently, in the absence of specific data pertaining to the follow-up of any extracoronary vascular abnormalities discovered in SCAD patients, management should mirror that of patients without SCAD.

For SCAD patient follow-up, coronary imaging could play a crucial role in determining the optimal duration of antiplatelet therapy for those with persistent dissection. Considering the risk of iatrogenic dissections, CTCA emerges as a potential alternative, albeit with limited current data.

The prognosis for SCAD patients is generally favorable, with the majority achieving complete recovery within 3 to 6 months following the index event. A US-based Mayo Clinic study estimated a 10-year survival rate of 92%, supported by a 94.4% 6-year survival rate from an Italian study. A Swiss series reported no deaths post the index event in 63 patients, a similar cohort size to a Japanese study that recorded just one death, while a Canadian series reported a 1–2% mortality rate at a 3.1-year follow-up. Notably, these studies also reported significant morbidity with major adverse cardiac events (MACE) occurring in 47.4% of the US series, 19.9% of the Canadian series, 37.8% of the Japanese series, and 14.6% of the Italian series [31].

High recurrence rates have been noted, with up to 30% at 4–10 years of follow-up in various series. Recurrences can be categorized into two distinct scenarios, one being an extension of the original lesion from the acute phase that hasn’t healed adequately, and the other being a de novo dissection occurring later (>30 days) and in different coronary segments. The latter scenario has been associated with a significant increase in SCAD recurrence rate [21]. After the initial event, cyclic chest pain, often heightened in the premenstrual period, is common. To accurately identify a recurrence, meticulous evaluation using ECG and troponin assay is recommended, with coronary study reserved exclusively for patients showing clear ischemic signs. The role of CTCA in ruling out SCAD recurrence remains uncertain. However, once ruled out, symptoms can be managed by mitigating vasospasm with vasodilator drugs, and in cases related to the menstrual cycle, with progestins [40].

For asymptomatic patients, angio-CT proves beneficial for follow-up, particularly in vessels <2.5 mm and cases not treated with angioplasty. Current data regarding the utility of cardiac MRI in these patients’ follow-ups are lacking [9].

Despite the potential benefits of beta-blockers and optimal blood pressure control, no therapeutic strategy to date has demonstrated a substantial reduction in recurrence rates. Consequently, the identification of possible risk factors for recurrent SCAD is a significant clinical objective. Severe coronary tortuosity has been identified as a potential risk marker for possible recurrence, although definitive evidence supporting this correlation is lacking. Whether tortuosity is a marker of underlying vasculopathy or a causative factor for arterial injury remains unclear [21].

Although physical activity has been frequently associated with SCAD recurrence, there is no substantial evidence to support such a correlation. On the contrary, numerous studies have highlighted the safety and benefits of cardiac rehabilitation in patients with SCAD. Given the physical and mental benefits of exercise, patients should be encouraged to resume full daily activities, inclusive of isometric activities and non-extreme sports. However, extreme endurance training, exhaustive exercise, elite competitive sports, or vigorous exertion at extreme environmental temperatures should be avoided [31]. Given the patients’ typically young age and lack of prior illnesses, SCAD often has a substantial emotional and psychological impact. Consequently, these patients have an elevated risk of developing post-traumatic stress disorder. Therefore, cognitive-behavioral or pharmacological therapies may prove beneficial [39].

## 4. Discussion

Since Wellens pattern was first described in 1982 by Chris de Zwaan, and Hein J. J. Wellens, recognition of this ECG abnormality has been of utmost importance because this syndrome represents a pre-infarction stage of severe stenosis of the proximal tract of the LAD artery that often progresses to a devastating anterior wall MI.

It is estimated that of all patients admitted with unstable angina, 14–18% present with this ECG pattern, thus it must be detected and treated promptly. Type A Wellens syndrome, characterized by biphasic T waves in leads V2 and V3, is the less common of the two (24%) while Type B Wellens syndrome, with deeply and symmetrically inverted T waves in V2–3, is the most frequent presentation (76%).

While initially thought to be distinct patterns, it is now uncertain whether type A and type B Wellens ECG changes represent different stages of the same phenomenon. In our case, we were able to promptly identify Wellens syndrome and thus the underlying life-threatening condition, and also document the transformation from a type A to a type B Wellens pattern through careful ECG monitoring. This finding supports the hypothesis that these patterns are not distinct entities, but rather different stages of the pathological changes associated with the coronary syndrome that ultimately led to LAD artery dissection.

The most frequent causes of proximal LAD artery dissection are atherosclerotic plaque, coronary artery vasospasm, and hypoxia resulting from increased cardiac demand. As of the time of writing, only one case of Wellens syndrome due to spontaneous LAD dissection has been reported.

Spontaneous LAD coronary artery dissection is a not so rare (up to 4%) cause of ACS and there are no typical ECG findings. CAG remains the ‘first-line’ examination in case of suspected ACS and is the gold standard for the diagnosis of SCAD.

In the case we presented, a 44-year-old female patient with no clear personal risk factors for coronary artery disease and no family history came to the emergency department (ED) with a history of exertional chest pain and subtle ECG findings that warranted deep clinical and ECG monitoring. Wellens ECG changes actually presented late in this patient, and prompt recognition of this pattern was essential and likely saved this patient’s life.

The typical chest pain associated with the ECG changes described, along with the increase in troponin values, suggest an ACS—an occurrence not typical in a young woman without major cardiovascular risk factors. Therefore, a coronary angiography was rapidly performed, which ruled out evidence of acute thrombotic occlusion, the main cause of ACS, and allowed for the diagnosis of a type 2 dissection of the LAD coronary artery. As a result, a proper and personalized treatment protocol was quickly implemented, preventing MI and reducing the risk of serious complications.

Treatment needs to be personalized to each patient’s specific conditions. Following admission, this patient experienced repeated episodes of chest tightness and pain; conservative treatment was chosen until her condition stabilized. However, if the patient is hemodynamically unstable or has persistent ischemia or recurrent dissection, PCI or CABG may be preferred.

Wellens syndrome due to LAD artery dissection is in fact a rare but potentially life-threatening condition that poses significant diagnostic challenges for healthcare providers.

This case outlines the importance of clinical and ECG monitoring and the critical role of the internal medicine specialist in the diagnostic process. The presentation of Wellens syndrome can be subtle and may require a high level of clinical suspicion, which is where internal medicine specialists can provide valuable input.

As well as cardiologists, internal medicine specialists are trained to recognize and manage a wide range of cardiovascular conditions, including ACS’s such as Wellens syndrome. They play a vital role in the early and non-invasive diagnosis and management of these conditions, particularly in patients with atypical presentations or underlying comorbidities.

However, cardiologists possess specialized knowledge and skills that are indispensable in providing comprehensive cardiac care, including the management of complex cases, and in guiding invasive diagnostic and therapeutic procedures when required.

The cooperation and coordination between these two specialities are pivotal in ensuring the best patient outcomes, as exemplified in our case of Wellens syndrome due to a spontaneous LAD coronary artery dissection.

## 5. Conclusions

In conclusion, spontaneous LAD coronary artery dissection causing Wellens syndrome, as seen in this case, is a particularly rare presentation. This makes the case noteworthy, not only for its rarity but also for its contribution to our understanding of Wellens syndrome. This case provides evidence that, in some patients, the different patterns of Wellens syndrome (Type A and Type B) may not be distinct entities, but rather different stages of the underlying pathological changes associated with the coronary syndrome—a finding that, to our knowledge, is unprecedented in medical literature.

Both these conditions can be challenging to diagnose. In fact, Wellens pattern typically appears during rest, in between episodes of chest pain; however, it can also change from one pattern to the other, either during episodes of chest pain or at rest, as observed in our case. This underscores the importance of ECG monitoring since misinterpretation of ECG findings can delay diagnosis, leading to significant morbidity and mortality. Cardiologists and internal medicine specialists play an essential role in diagnosing and managing Wellens syndrome. Their synergistic expertise can be critical in promptly identifying this condition and ensuring timely intervention to prevent further complications.
